# The role of domestic reservoirs in domestically acquired *Salmonella* infections in Norway: epidemiology of salmonellosis, 2000–2015, and results of a national prospective case–control study, 2010–2012

**DOI:** 10.1017/S0950268818002911

**Published:** 2018-11-15

**Authors:** E. MacDonald, R. White, R. Mexia, T. Bruun, G. Kapperud, L. T. Brandal, H. Lange, K. Nygård, L. Vold

**Affiliations:** 1 Department of Zoonotic, Food- and Waterborne infections, Norwegian Institute of Public Health, Oslo, Norway; 2 Department of Infectious Disease Epidemiology and Modelling, Norwegian Institute of Public Health, Oslo, Norway; 3 Department of Vaccine Preventable Diseases, Norwegian Institute of Public Health, Oslo, Norway

**Keywords:** Food-borne infections, *Salmonella*, salmonellosis, surveillance

## Abstract

In Norway, incidence of sporadic domestically acquired salmonellosis is low, and most frequently due to *Salmonalla* Typhimurium. We investigated the risk factors for sporadic *Salmonella* infections in Norway to improve control and prevention measures. Surveillance data for all *Salmonella* infections from 2000 to 2015 were analysed for seasonality and proportion associated with domestic reservoirs, hedgehogs and wild birds. A prospective case–control study was conducted from 2010 to 2012 by recruiting cases from the Norwegian Surveillance System for Communicable Diseases and controls from the Norwegian Population Registry (389 cases and 1500 controls). Univariable analyses using logistic regression were conducted and a multivariable model was developed using regularised/penalised logistic regression. In univariable analysis, eating snow, dirt, sand or playing in a sandbox (aOR 4.14; CI 2.15–7.97) was associated with salmonellosis. This was also the only exposure significantly associated with illness in the multivariable model. Since 2004, 34.2% (*n* = 354) of *S.* Typhimuirum cases had an MLVA profile linked to a domestic reservoir. A seasonal trend with a peak in August for all *Salmonella* types and in February for *S.* Typhimurium was observed. Indirect exposure to domestic reservoirs remains a source of salmonellosis in Norway, particularly for children. Information to the public about avoiding environmental exposure should be strengthened and initiatives to combat salmonellosis in the food chain should be reinforced.

## Introduction

Salmonellosis is the second most frequently reported bacterial food- and water-borne infection after campylobacteriosis in many European countries, including Norway [1]. In 2014, the incidence rate of confirmed salmonellosis was 23.4 cases per 1 00 000 population in the European Union (EU)/European Economic Area (EEA), marking a statistically significant decrease in salmonellosis in Europe in the 7-year period from 2008 [2]. Similar trends have been observed in Norway, where salmonellosis has been notifiable to the Norwegian Surveillance System for Communicable Disease (MSIS) since 1975. In the 1980s, incidence of salmonellosis in Norway increased significantly, a trend seen in other European countries [3, 4]. This increase was primarily due to the emergence of *Salmonella* Enteritidis infection in Europe, which was being acquired by Norwegians while traveling, particularly in the context of increased charter tourism [5]. However, since 2009 the national incidence of salmonellosis has decreased substantially, due to a parallel reduction in imported cases of *S.* Enteritidis, which has been attributed to successful control programmes in poultry and eggs in the EU [1].

In Europe, the most common serovars are *Salmonella enterica* serovar Enteritidis (*S*. Enteritidis), which accounted for 41.3% of reported cases in 2012, followed by *S. enterica* serovar Typhimurium (*S.* Typhimuirum), excluding monophasic *S.* Typhimurium 1,4,[5],12:i:-, which accounted for 22.1% of reported cases [1]. The natural reservoirs for *Salmonella* include a wide range of wild and domesticated animals, which vary depending on the specific *Salmonella* serovar implicated [6]. Food products associated with infection also depend on the implicated serovar [7]. *S.* Enteritidis infections have most frequently been associated with consumption of poultry and eggs [6], while *S*. Typhimurium has been linked to a wide range of products, including beef, pork, chicken and contact with animals. In addition to food-borne transmission [8], other exposures have been linked to infections including foreign travel [9–12], drinking untreated water [13], contact with animals such as farm animals and pets (including herptiles) [14–16], and contact with pet feed [17, 18].

**Fig. 1.**
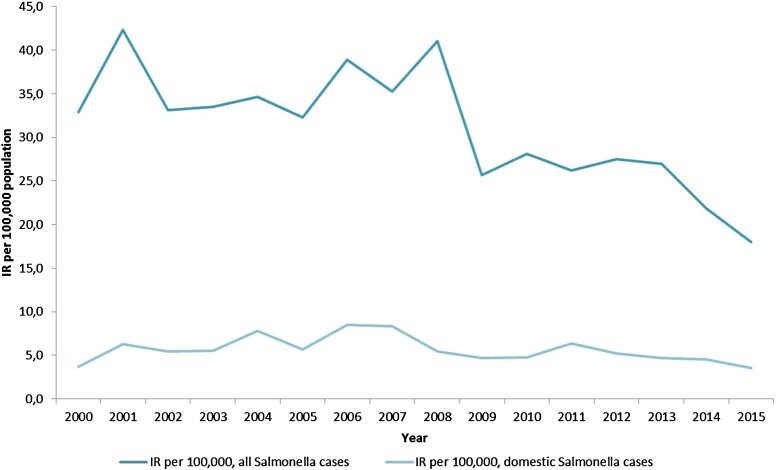
Annual incidence of salmonellosis per 1 00 000 population by geographical location of infection, Norway, 2000–2015*. *Excluding *S.* Typhi and *S.* Paratyphi.

Between 2000 and 2015, 23 546 cases of salmonellosis were reported to MSIS, corresponding to an average annual incidence rate of 31.1 cases per 100 000 population during that period (Fig. 1). Between 70% and 80% of all salmonellosis cases reported annually are associated with travel abroad [26], corresponding to an annual average incidence rate of 5.7 domestically acquired cases per 100 000 population. The low incidence of sporadic salmonellosis is primarily due to the negligible levels of *Salmonella* in Norwegian livestock and food [19]. The results from *Salmonella* surveillance in Norway has consistently shown that Norwegian cattle, swine and poultry populations are rarely infected with *Salmonella*, with an estimated prevalence below 0.05% in the examined populations since surveillance programmes were established [19]. This is supported by the results of a previous case–control study in Norway conducted in 1994 which did not demonstrate any association between illness and consumption of domestically produced red meat, eggs or poultry [20]. Given the low levels of *Salmonella* in livestock in Norway, Norway is granted additional guarantees when importing live animals, feed and food products of animal origin from the EU. Despite this, previous outbreaks of salmonellosis identified in Norway have been linked to a wide range of products, including meat [21–23], fresh produce [24–26] and other food items [27, 28], almost all of which were imported.

There are two known domestic reservoirs for *Salmonella* in Norway, both of which harbour *S.* Typhimurium: wild birds [13, 29, 30] and hedgehogs [31, 32]. Strains associated with these reservoirs have been implicated in earlier outbreaks [33] and evidence from environmental investigations [33] and a case–control study [13] document the existence of reservoirs and transmission of distinct *S.* Typhimurium strains from these two animal hosts. Genetic profiles discerning these reservoirs have been identified through genotyping of *S*. Typhimurium isolates by multiple-locus variable-number tandem-repeat analysis (MLVA) at the National Reference Laboratory for Enteropathogenic Bacteria, which has been done on all human isolates of *S*. Typhimurium received since April 2004.

As a national case–control study to identify risk factors for domestically acquired salmonellosis has not been conducted for more than 20 years, it was desirable to reassess the impact of livestock, poultry and eggs on indigenous salmonellosis. Additionally, it was necessary to document the extent to which domestic wildlife reservoirs contribute to the current burden of salmonellosis in Norway and potentially identify new exposures that can explain indigenous infection. Therefore, the main objectives of this study were to: (1) describe the epidemiology of salmonellosis caused by serovars associated with domestic reservoirs, including the seasonality, and (2) investigate risk factors for sporadic indigenous salmonellosis in Norway in order to identify areas where control and prevention measures could be improved.

## Methods

### Epidemiology of domestic salmonellosis

Human cases of salmonellosis are notifiable to MSIS by both clinicians and laboratories. For each notified case, information on travel abroad has been recorded since 1982, with improved information on likely country of infection available since 1995. Cases are classified as infected abroad if the case was abroad during the incubation period. All isolates received by the National Reference Laboratory are routinely serotyped and *S*. Typhimurium and *S*. Enteritidis isolates are routinely genotyped by MLVA since April 2004 and 2014, respectively. The hedgehog strains largely fit the MLVA profile 3-15-NA-NA-311 of *S.* Typhimurium, with some variations occurring at the second locus. For wild birds, several MLVA profiles of *S.* Typhimurium have been identified (including 2-13-3-NA-212, with single locus variations, 2-10-6-NA-212 and 2-13-4-NA-212), which differ from the hedgehog MLVA profile.

To describe the epidemiology of domestically acquired salmonellosis (except typhoid fever and paratyphoid fever), we calculated the proportion of cases by serovar (*S.* Typhimurium *vs.* other serovars) between 2000 and 2015 and the proportion of cases due to *S.* Typhimurium with an MLVA profile associated with either wild birds or hedgehogs since 2004. Patterns of seasonality were examined for all salmonellosis cases irrespective of serovar, and then separately for *S.* Typhimurium and all other salmonella serovars reported from 2000 to 2015. Negative binomial regression was used to investigate month and year discreteness using ‘month of illness onset’ as exposure with January as the reference month. An IRR>1 indicated higher risk while an IRR <1 indicated a lower risk.

### Case–control study design

A national prospective case–control study of exposures associated with campylobacteriosis and salmonellosis was conducted from July 2010 to September 2012. This article presents the results of the salmonellosis study as results for campylobacteriosis have been previously published [34]. A case was defined as a resident of Norway with laboratory-confirmed salmonellosis caused by any serovar (except Typhi and Paratyphi) reported to MSIS from July 2010 to September 2012. All cases reported to MSIS during the study period with postal address available were included in the study. Controls were randomly selected from the Norwegian Population Registry, a continuously updated registry of all residents of Norway. Four hundred unmatched controls were selected on a monthly basis. Cases and controls were mailed a paper questionnaire and a cover letter with a prepaid return envelope. If a case or control was under the age of 16, the questionnaire was sent to a parent who was asked to help the child complete the questions. Participants were asked to return the questionnaire by mail, or answer the questions online using an electronic version of the questionnaire, or through a telephone interview. Submission of the completed questionnaire was considered informed consent for participation. This study received approval from the Regional Ethical Committee for South East Norway.

### Exposures

Fifty-eight broad yes/no questions concerning potential risk factors were asked in the questionnaire, mostly concerning exposures in the preceding week (e.g. ‘During the last week, did you eat chicken?’). If respondents answered affirmatively, they were requested to answer derivative questions about exposure frequency and further details about their exposure (e.g. ‘Was the purchased chicken raw and frozen?’ and ‘Was the chicken purchased ready-made?’). For data cleaning purposes, ‘uncertain’ responses were coded as missing.

### Confounders

We considered sex (male/female), age (in years), number of household members, county (19 counties) and education (not completed primary school, primary to middle school, high school, university and other) as potential confounders. Age and number of household members were specified as continuous variables to satisfy the requirements for a parsimonious model, as manipulation of continuous variables can inadvertently lead to a biased model. After model fitting, residuals were assessed to ensure linearity assumptions were not violated.

### Statistical analyses

Adjusting for the above listed potential confounders, we ran 58 separate logistic regression analyses on the broad risk factors, accounting for multiple testing by using the Bonferroni correction. We then ran supplemental logistic regression analyses in the derivative risk factor questions. When the questions were about exposure frequency, the entire cohort was analysed. When the questions were about the derivative exposures, the analysis was restricted to only those who answered yes to the original broad risk factor. Controls were used as the comparison group for all analyses. We also conducted stratified analysis by serovar (*S.* Typhimurium *vs.* all other *Salmonella* serovars combined) in order to investigate whether different risk factors were present for cases attributable to known domestic reservoirs.

We then constructed a multivariable model to investigate the associations between broad risk factors and any *Salmonella* infection. We included all 58 broad risk factors (plus potential confounders) as explanatory variables in our multivariable model. Due to the large number of explanatory variables, we used BOLASSO to construct a parsimonious model [24]. A complete description of the methodology has been previously published [34]. Separate models were created for all *Salmonella* serovars, *S.* Typhimurium and all non-*S.* Typhimurium infections.

## Results

### Epidemiology of domestically acquired salmonellosis

**Fig. 2.**
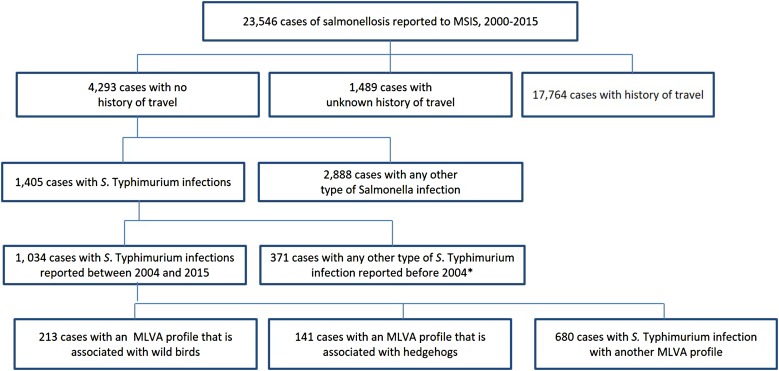
Cases of salmonellosis^#^ reported to MSIS linked to known domestic reservoirs, Norway, 2000–2015. ^#^Excluding *S.* Typhi and *S.* Paratyphi. *MLVA data only available from April 2004.

Between 2000 and 2015, 23 546 cases of salmonellosis were reported to MSIS. Of these, 18.2% (*N* = 4293) reported not having travelled abroad during the incubation period. Among cases that were not travel-related, *S.* Typhimurium was the most commonly identified serovar, accounting for 32.7% (*n* = 1405) of domestic cases (Fig. 2). Since MLVA started being routinely used in 2004, 1034 cases were identified with domestically acquired *S.* Typhimurium infections. Of these, 13.6% (*n* = 141) cases had the hedgehog MLVA profiles and 20.6% (*n* = 213) cases had the wild bird MLVA profiles.

Salmonellosis incidence for all indigenous cases (*n* = 4293) exhibited two peaks, one small peak in February (IRR 1.12, *P* = 0.421), though not significant, and a large peak in the summer, in particular August (IRR 2.23, *P* = 0.000). For *S.* Typhimurium (*n* = 1405), there was a large peak in February (IRR 2.19, *P* = 0.028) and a large peak in August (IRR 4.08, *P* = 0.000). For all other *Salmonella* types, there was a large peak in August (IRR 4.17, *P* = 0.000). The number of cases associated with specific MLVA profiles attributable to either wild birds or hedgehogs were insufficient to test for trend.

### Case–control study population

During the 27-month study, 3196 cases of salmonellosis were notified via MSIS. Of these, 2058 cases were not initially reported as infected abroad or had missing travel information, had valid Norwegian postal addresses and were sent the questionnaire. Responses to the questionnaire were received from 1190 cases (58%). Questionnaires were sent to 5808 controls, of which 1738 responded (30%). We subsequently excluded 801 cases and 110 controls who had been outside of Norway in the previous 2 weeks, and 128 controls with unspecified gastrointestinal illnesses, leaving 389 cases and 1500 controls, for a total of 1889 subjects. When the enrolled controls were compared with the Norwegian population, males were under-represented (44.5% of controls compared with 50.3% of the population, *P* = 0.002) and study participants were older than the general population (*P* < 0.001).

### Description of cases

Of the 389 cases enrolled, 25.7% (100 cases) were infected with *S.* Typhimurium, excluding the monophasic variant. The remaining cases were infected with monophasic *S.* Typhimurium (*n* = 35), *S.* Enteritidis (*n* = 57), *Salmonella* with an unspecified serovar affiliation (*n* = 107), or any other serovars other than Typhimurium or Enteritidis (*n* = 90). The mean age of cases and controls was 40.9 years (95% CI 39.6–42.1) and 42.7 years (95% CI 21.6–43.8), respectively. Male subjects comprised 44.8% of cases and 44.5% of controls. Cases belonged to larger households and reported higher education levels than controls (Table 1). There were relatively more cases from western Norway (24.7% of cases compared with 16.0% of controls) and controls from the Oslo area (11.6% of cases compared with 25.4% of controls).

**Table 1.** Demographic characteristics of cases (*n* = 389) and controls (*n* = 1500)^a^

Variable	All *Salmonella* cases (*n* = 389) *n* (%)	*Salmonella* Typhimurium cases^b^ (*n* = 100) *n* (%)	Non-*Salmonella* Typhimurium cases (*n* = 289) *n* (%)	Controls (*n* = 1500) *n* (%)	*P* value^c^
Sex					
Male	171 (44.8)	45 (46.4)	126 (44.2)	662 (44.5)	
Female	211 (55.2)	52 (53.6)	159 (55.8)	824 (55.5)	0.940
Age group					
0–9	73 (19.8)	32 (34.)	41 (15.0)	147 (10.0)	
10–19	28 (7.6)	8 (8.5)	20 (7.3)	138 (9.4)	
20–39	70 (19.0)	21 (22.3)	49 (17.9)	316 (21.6)	
40–59	108 (29.3)	18 (19.1)	90 (32.8)	493 (33.7)	
60–94	89 (24.2)	15 (16.0)	74 (27.0)	369 (25.2)	<0.001
Number of household members					
Live alone	50 (13.0)	8 (8.2)	42 (14.7)	195 (13.1)	
Two people	126 (32.8)	21 (21.4)	105 (36.7)	531 (35.7)	
Three to five people	190 (49.5)	58 (59.2)	132 (46.2)	704 (47.4)	
Six or more people	18 (4.7)	11 (11.2)	7 (2.4)	56 (3.8)	0.642
Education level completed					
Primary school not completed	71 (19.3)	31 (33.3)	40 (14.6)	233 (15.8)	
Primary school or middle school	83 (22.6)	14 (15.1)	69 (25.2)	222 (15.1)	
High school	125 (34.1)	27 (29.0)	98 (35.8)	465 (31.5)	
University	77 (21.0)	19 (20.4)	58 (21.2)	499 (33.9)	
Other	11 (3.0)	2 (2.2)	9 (3.3)	55 (3.7)	<0.001
Geographical region^d^					
Oslo and Akershus	45 (11.6)	10 (10.0)	35 (12.1)	381 (25.4)	
Hedmark and Oppland	16 (4.1)	4 (4.0)	12 (4.2)	112 (7.5)	
Eastern Norway	83 (21.3)	12 (12.0)	71 (24.6)	303 (20.2)	
Agder and Rogaland	68 (17.5)	14 (14.0)	54 (18.7)	194 (12.9)	
Western Norway	96 (24.7)	43 (43.0)	53 (18.3)	240 (16.0)	
Trøndelag	43 (11.1)	9 (9.0)	34 (11.8)	126 (8.4)	
Northern Norway	38 (9.8)	8 (8.0)	30 (10.4)	144 (9.6)	<0.001

a Totals may not add due to missing data.

b Excluding monophasic variant.

c Comparing controls and all *Salmonella* cases.

d Counties are grouped in the following categories: Eastern Norway (Telemark, Buskerud, Vestfold and Østfold), Western Norway (Møre og Romsdal, Sogn og Fjordane and Hordaland), Trøndelag (Nord−Trøndelag and Sør−Trøndelag) and Northern Norway (Finnmark, Troms and Nordland).

### Univariable analysis

After correction for multiple testing and adjusting for confounders, we found that eating snow, icicles, sand, dirt or playing in a sandbox significantly increased the odds of salmonellosis among all cases (Table 2). The full univariable results are available in the Supplementary material (Table S1). Consumption of red meat, poultry or eggs was not associated with illness. Contact with hedgehogs, wild birds or reptiles was not significantly associated with salmonellosis, but <1% of cases and controls reported such exposures. When stratified by serovar, none of the exposures was significant for non-Typhimurium cases. For *S.* Typhimurium cases, the ORs for eating snow, icicles, sand, dirt or playing in a sandbox, attending/working in a kindergarten/nursery and eating food prepared in a kindergarten/nursery increased. When stratified by age, eating snow, dirt, sand or playing in a sandbox did not remain significant for adults or children, although the aOR for children increased to 3.55 (CI 0.73–17.27). There were too few cases to stratify by both age and *Salmonella* serovar.

**Table 2.** Exposures associated with *Salmonella* infection in univariable analysis by serovar^a^

	All *Salmonella* (*n* = 389)		*S. Typhimurium* only (*n* = 100)		*S.* non-*Typhimurium* only (*n* = 289)		Controls (*n* = 1500)
Exposure	*n* (%) exposed	aOR	*n* (%) exposed	aOR	*n* (%) exposed	aOR	*n* (%) exposed
Eaten snow, icicle, sand, dirt or played in sandbox	52 (15)	4.14 (2.15, 7.97)***	26 (30)	6.70 (2.25, 19.94)*	26 (10)	2.88 (1.29, 6.41)	87 (6)
Eating undercooked meat	34 (11)	1.77 (1.16, 2.71)	9 (11)	2.00 (0.91, 4.38)	25 (11)	1.68 (1.04, 2.72)	119 (9)
Attend or work in a kindergarten or nursery	65 (17)	1.62 (1.07, 2.46)	30 (31)	2.42 (1.28, 4.59)	35 (12)	1.24 (0.74, 2.06)	135 (9)
Dog in household	92 (25)	1.44 (1.07, 1.93)	24 (25)	1.59 (0.91, 2.75)	68 (25)	1.42 (1.02, 1.98)	253 (19)
Drinking purchased bottled water	114 (36)	1.44 (1.07, 1.93)	29 (33)	1.22 (0.71, 2.11)	85 (38)	1.57 (1.13, 2.17)	418 (30)
Cat in household	98 (26)	1.38 (1.03, 1.86)	31 (32)	1.47 (0.86, 2.50)	67 (24)	1.38 (0.99, 1.92)	268 (19)
Eating food made in a kindergarten, or nursery	59 (47)	1.19 (0.65, 2.20)	27 (68)	2.48 (0.90, 6.87)	32 (37)	0.81 (0.39, 1.70)	118 (33)
Contact with cat or cat feces	85 (29)	1.19 (0.88, 1.63)	27 (38)	1.57 (0.89, 2.77)	58 (26)	1.08 (0.76, 1.54)	298 (24)

a All variables where CI does not contain 0 for at least one *Salmonella* serovar; answers for all variables were not available from all participants; aOR: adjusted for sex, age, number of people in household, county (dummy) and education; corrected for multiple testing.

Significance indicators: *0, ***0.001, **0.01.

In univariable analysis, having a cat in the household was associated with illness for all salmonellosis cases (aOR 1.38, CI 1.03–1.86). Having a dog in the household was associated with illness for all salmonellosis cases (aOR 1.44, CI 1.07–1.93) and for non-*S*. Typhimurium cases (aOR 1.42, CI 1.02–1.98). When stratified by age, living in a household with a cat and having a dog in the household remained associated with salmonellosis for both children and adults, although this was much lower for adults. In addition to having a cat in the household, contact with cats/cat faeces was marginally associated with salmonellosis for children (OR 3.9), although this was not associated with illness for adults.

In univariable analysis, consumption of chicken, turkey, mutton/lamb and beef were not significantly related to illness. However, further inspection of the derivative results for chicken and turkey demonstrated that among chicken-eaters, eating ready-made chicken from a commercial kitchen was significantly associated with higher odds of salmonellosis (OR 2.34, 95% CI 1.58–3.48). Likewise, within turkey-eaters, eating products such as sausages and meatballs was significantly associated with increased risk (OR 10.32, 95% CI 1.17–90.92). We also found a significant dose–response association with number of times eating ready-made chicken bought from a commercial kitchen (OR 1.47, 95% CI 1.22–1.75), and number of times eating turkey products such as sausages and meatballs (OR 4.74, 95% CI 1.11–20.22). Among people who did not eat meat well done, eating raw, rare or undercooked chicken/turkey (OR 2.82, 95% CI 0.91–8.71) and mutton/lamb (OR 7.18, 95% CI 0.79–64.93) were associated with higher odds of salmonellosis but were not statistically significant.

A number of exposures were found to significantly decrease the odds of salmonellosis, including consumption of fresh herbs, dried herbs, soft cheese, lettuce and raw vegetables (Table 3). Consumption of foreign bought meat and cured meats was also associated with reduced risk of illness. Among pork eaters, purchasing raw unfrozen pork was associated with decreased odds of salmonellosis (OR 0.66, 95% CI 0.44–0.97). Consumption of herbs, pork, lettuce and raw vegetables remained protective for both *S.* Typhimurium and all other *Salmonella* types combined when stratified by *Salmonella* serovar.

**Table 3.** Exposures associated with reduced risk of *Salmonella* infection in univariable analysis by serotype^a^

	All *Salmonella* (*n* = 389)		*S.* Typhimurium only (*n* = 100)		*S.* non-Typhimurium only (*n* = 289)		Controls
Exposure	*n* (%) exposed	aOR	*n* (%) exposed	aOR	*n* (%) exposed	aOR	*n* (%) exposed
Eating dried herbs	203 (65)	0.45 (0.33, 0.61)***	51 (65)	0.39 (0.22, 0.67)*	152 (65)	0.47 (0.34, 0.65)***	1113 (81)
Eating fresh herbs	45 (14)	0.48 (0.33, 0.69)**	13 (15)	0.46 (0.23, 0.91)	32 (14)	0.47 (0.31, 0.71)*	409 (30)
Eating pork	138 (46)	0.48 (0.37, 0.64)***	34 (43)	0.44 (0.26, 0.73) +	104 (47)	0.50 (0.36, 0.68)***	863 (63)
Eating lettuce	198 (60)	0.51 (0.38, 0.68)***	48 (59)	0.44 (0.25, 0.77)	150 (60)	0.52 (0.38, 0.72)**	1053 (76)
Eating raw vegetables	232 (71)	0.59 (0.43, 0.80)*	55 (67)	0.53 (0.29, 0.94)	177 (72)	0.62 (0.44, 0.88)	1159 (83)
Eating cured meats	164 (50)	0.60 (0.46, 0.78)**	53 (61)	0.95 (0.57, 1.58)	111 (46)	0.51 (0.38, 0.69)***	879 (64)
Eating meat purchase abroad	53 (15)	0.61 (0.42, 0.88)	12 (13)	0.54 (0.24, 1.21)	41 (16)	0.62 (0.41, 0.92)	324 (24)
Eating ground meat	270 (82)	0.62 (0.43, 0.89)	78 (91)	0.99 (0.44, 2.19)	192 (78)	0.57 (0.39, 0.84)	1206 (87)
Eating soft cheese	65 (19)	0.63 (0.46, 0.87)	14 (15)	0.60 (0.31, 1.14)	51 (21)	0.63 (0.44, 0.90)	464 (34)
Eating mutton/lamb	46 (13)	0.72 (0.50, 1.03)	7 (8)	0.42 (0.18, 1.02)	39 (16)	0.81 (0.55, 1.19)	278 (20)
Contact with wild birds or bird feces	18 (5)	0.74 (0.43, 1.25)	8 (10)	1.83 (0.81, 4.14)	10 (4)	0.50 (0.26, 0.99)	115 (8)
Drinking tapwater at home	359 (95)	0.77 (0.40, 1.46)	97 (99)	2.68 (0.35, 20.73)	262 (94)	0.62 (0.32, 1.21)	1362 (97)
Eating raw berries	127 (37)	0.87 (0.67, 1.13)	43 (48)	1.24 (0.77, 2.01)	84 (33)	0.77 (0.57, 1.03)	601 (44)

a All variables where CI does not contain 0 for at least one *Salmonella* serovar; answers for all variables were not available from all participants; adjusted for sex, age, number of people in household, county (dummy) and education; corrected for multiple testing.

Significance indicators: *0, ***0.001, **0.01.

### Multivariable analysis

When placed in the same model, the only exposure that was significantly associated with salmonellosis for all cases was eating snow, icicles, sand, dirt or playing in sandboxes (OR 1.22). In multivariable models which included only *S.* Typhimurium cases or only non-*S.* Typhimurium cases, none of the exposures was associated with illness.

## Discussion

Domestically acquired salmonellosis remains uncommon and commonly reported exposures, such as pork, poultry and eggs, are not significantly associated with *Salmonella* infections in Norway. The results of this study reinforce previous findings and indicate that the efforts invested to combat *Salmonella* in Norway during the past century have been successful. While only 34% of cases of salmonellosis due to *S*. Typhimurium between 2004 and 2015 could be linked to domestic reservoirs through MLVA genotyping, the results of the case–control study support that indirect or environmental exposure remain sources of infection for salmonellosis in Norway. Although neither direct contact with wild birds nor hedgehogs was associated with illness, <1% of respondents reported direct or indirect contact with either, suggesting that environmental exposure or unknown indirect contact with domestic reservoirs may be underestimated by this study. Eating dirt, sand, snow, ice or playing in sandboxes was associated with infection in multivariable analysis, which supports that environmental exposure to *Salmonella* may present a risk particularly for children.

The role of Norway's domestic reservoirs in transmitting *Salmonella*, hedgehogs and wild birds is well documented. Wild hedgehogs have been found to carry *Salmonella* in Norway [31–33], as well as in other countries, including Denmark [35] and the UK [36]. Faecal carriage studies have demonstrated that hedgehogs in areas in Norway with a history of outbreaks (western and southeastern Norway) carried *S.* Typhimurium. Wild birds are also a known reservoir for *S.* Typhimurium in Norway, most frequently found in passerines, mainly in bull finches and greenfinches, although the serovar has also been isolated from gulls, waterfowl, birds of prey and doves [30]. Gulls are frequently carriers of *Salmonella*, with Typhimurium being the predominant serovar [33]. However, the genotypes found in gulls are different from those among passerines, and are rarely detected in human patients [29]. Contact with wild birds has been reported as an exposure associated with salmonellosis in previous studies in Norway, including a 1990–1992 case–control study which concluded that winter feeding of birds as well as activities linked to bird-feeding including cleaning bird feeders, tending sick birds and eating snow under bird feeders could be linked to bird strains of *S*. Typhimurium [13].

Exposure to these reservoirs may also be exacerbated by having a cat or dog in the household. Cats and dogs can serve as asymptomatic carriers and both have been previously associated with salmonellosis [37]. Cats are effective predators of wild birds, and the genotypes linked to the passerine reservoir are sporadically detected in cats at the Norwegian Veterinary Institute [38]. As dogs are coprophagous, they are also prone to exposure through contact with faecal matter or other detritus. Children that consume dirt or snow may be unknowingly exposing themselves to cat or dog faeces. In Norway, most children attend kindergarten or nursery where daily outdoor activities are ubiquitous, presenting an additional opportunity for environmental exposure to *Salmonella*. A previous study from the Netherlands also found a positive association between sandboxes and *S.* Typhimurium in children with a population-attributable fraction (PAF) of 14% and PAF of 32% for *S.* Typhimurium DT104 [39]. Families and childcare facilities should therefore make extra effort to ensure that children do not consume dirt, sand or snow, and that sandboxes are routinely covered.

The results of this study also support that the epidemiology of *S.* Typhimurium infections may differ from other *Salmonella* types, leading to differences in risk factors by serovar. This is not unexpected as salmonellae of different serovars exhibit different host preferences and are consequently associated with different products, although there is a considerable overlap. When analysing *Salmonella* serovars other than *S*. Typhimurium, none of the exposures was associated with infection. This may be due to the fact that this category contains a range of serovars which may be associated with many different exposures. However, exposures that were associated with reduced odds of infection were relatively similar for all *Salmonella* serovars, suggesting that these exposures may be reflective of healthy dietary patterns and that children, who are predisposed to environmental infection, are less likely to consume these food items. The variability in the epidemiology of *S.* Typhimurium *vs.* all other *Salmonella* serovars combined is reinforced by the examination of seasonality. The trend identified in domestically acquired cases reported from 2000 to 2015 with a peak in the summer months is consistent for all types of *Salmonella* infection, mirroring the trends observed in many other European countries. This is most likely due to secondary transmission from individuals with salmonellosis infections returning from travel abroad to countries with greater incidence. Warmer temperatures leading to potential food handling errors and increases in known risk activities such as barbecuing and gardening may also contribute to this peak [40]. The less-pronounced peak in the winter months in cases of *S.* Typhimurium infection is more likely to be linked to the presence of domestic reservoirs. A previous analysis of the epidemiology of salmonellosis in Norway between 1966 and 1996 found that the majority of human and avian cases of *S.* Typhimurium infections, with the discrete phenotypic and genotypic characteristics of the passerine reservoir, were reported between the months of January through April [13].

There are several limitations to this study that must be considered. Only laboratory-confirmed cases were included in the study. It is possible that milder cases did not seek healthcare, which could hide patterns of risk factors. In addition, small numbers of cases infected with specific *Salmonella* serovars prevented stratified analysis beyond *S.* Typhimurium and it is possible that the role of specific exposures may have been obscured due to the grouped analysis. Despite efforts to increase the response proportion through reminders, only 58% of cases and 30% of controls participated in the study, which could mean that results are not necessarily representative of the whole Norwegian population. Additionally, the possibility of recall bias cannot be excluded. The delay between illness onset and interview may have introduced a recall bias, leading to underestimation of risk factors susceptible to recall problems. Conversely, cases may have had a better recollection of exposures than controls and may have been more likely to report consumption of products typically known to be associated with gastroenteritis. Efforts were made to minimise this bias by sending out questionnaires to both cases and controls on an ongoing basis. However, some segments of the population may have been less likely to have been reached via postal address, which is an inherent limitation of the data collection method. This may have led to an under-representation of especially mobile groups, such as students or immigrant populations. In addition, the questionnaire was only available in Norwegian, which may also have led to under-representation of the non-Norwegian-speaking population.

## Conclusions

Norway continues to have a low incidence of salmonellosis as few reservoirs are present in the country. However, there is evidence that indirect contact with domestic reservoirs, small birds and hedgehogs, through environmental exposure remains a source of infection, particularly for children. Children should be encouraged to avoid eating snow, dirt or sand, and parents and childcare providers should ensure sandboxes are covered when not in use to avoid contamination with animal faeces. The importance of good hand hygiene should not be underestimated, especially after having contact with domestic or wild animals, and playing or working outdoors. In addition, efforts to combat salmonellosis on all stages in the food chain should be reinforced, on both national and international levels.

## Supplementary material

For supplementary material accompanying this paper visit https://doi.org/10.1017/S0950268818002911. click here to view supplementary material
