# Supplementary material for: The role of domestic reservoirs in domestically acquired Salmonella infections in Norway: epidemiology of salmonellosis, 2000–2015, and results of a national prospective case–control study, 2010–2012
Source: Epidemiol Infect. 2018 Nov 15;147:e43. doi: 10.1017/S0950268818002911 (PMC6518537; doi:10.1017/S0950268818002911)
Supplement: Supplementary file 1 [file S0950268818002911sup001.docx]

**Supplementary table 1. Complete univariable results of case-control study by *Salmonella* serotype^#^**

|  | **All *Salmonella***  **(n=389)** | | | ***S. Typhimurium* only**  **(n=100)** | | | **S. non-Typhimurium only**  **(n=289)** | | | **Controls**  **N=1500** |
| --- | --- | --- | --- | --- | --- | --- | --- | --- | --- | --- |
| **Exposure** | **n (%)**  **exposed** | **Crude OR** | **aOR** | **n (%)**  **exposed** | **Crude OR** | **aOR** | **n (%)**  **exposed** | **Crude OR** | **aOR** | **n (%)**  **exposed** |
| Eaten snow, icicle, sand, dirt or played in sandbox | 52 (15) | 2.67 (1.85, 3.85)*** | 4.14 (2.15, 7.97)** | 26 (30) | 6.53 (3.94, 10.84)*** | 6.70 (2.25, 19.94)* | 26 (10) | 1.68 (1.06, 2.66) | 2.88 (1.29, 6.41) | 87 (6) |
| Attend or work in a kindergarten or nursery | 65 (17) | 2.06 (1.49, 2.84)*** | 1.62 (1.07, 2.46) | 30 (31) | 4.40 (2.77, 7.01)*** | 2.42 (1.28, 4.59) | 25 (11) | 1.34 (0.85, 2.11) | 1.68 (1.04, 2.72) | 135 (9) |
| Eat food made in a kindergarten or nursery | 59 (47) | 1.80 (1.19, 2.72) | 1.19 (0.65, 2.20) | 27 (68) | 4.24 (2.11, 8.52)** | 2.48 (0.90, 6.87) | 35 (12) | 1.41 (0.95, 2.10) | 1.24 (0.74, 2.06) | 118 (33) |
| Fed or had contact with hedgehog | 3 (1) | 1.62 (0.42, 6.29) | 1.60 (0.38, 6.64) | 1 (1) | 2.19 (0.27, 18.07) | 2.53 (0.26, 24.84) | 2 (1) | 1.43 (0.30, 6.93) | 1.46 (0.28, 7.59) | 7 (1) |
| Contact with turtle, snakes, or other reptiles | 3 (1) | 1.62 (0.42, 6.29) | 1.60 (0.38, 6.64) | 1 (1) | 2.19 (0.27, 18.07) | 2.53 (0.26, 24.84) | 2 (1) | 1.43 (0.30, 6.93) | 1.46 (0.28, 7.59) | 7 (1) |
| Cat in household | 98 (26) | 1.49 (1.14, 1.94) | 1.38 (1.03, 1.86) | 31 (32) | 1.97 (1.26, 3.09) | 1.47 (0.86, 2.50) | 85 (38) | 1.40 (1.04, 1.87) | 1.57 (1.13, 2.17) | 268 (19) |
| Dog in household | 92 (25) | 1.47 (1.12, 1.93) | 1.44 (1.07, 1.93) | 24 (25) | 1.46 (0.90, 2.37) | 1.59 (0.91, 2.75) | 68 (25) | 1.47 (1.08, 2.00) | 1.42 (1.02, 1.98) | 253 (19) |
| Eating undercooked meat | 34 (11) | 1.35 (0.90, 2.01) | 1.77 (1.16, 2.71) | 9 (11) | 1.37 (0.67, 2.81) | 2.00 (0.91, 4.38) | 67 (24) | 1.34 (0.99, 1.82) | 1.38 (0.99, 1.92) | 119 (9) |
| Drinking purchased bottled water | 114 (36) | 1.33 (1.03, 1.72) | 1.49 (1.12, 1.99) | 29 (33) | 1.16 (0.73, 1.84) | 1.22 (0.71, 2.11) | 58 (26) | 1.13 (0.81, 1.56) | 1.08 (0.76, 1.54) | 418 (30) |
| Contact with cat or cat feces | 85 (29) | 1.29 (0.97, 1.72) | 1.19 (0.88, 1.63) | 27 (38) | 1.90 (1.16, 3.11) | 1.57 (0.89, 2.77) | 32 (37) | 1.21 (0.74, 1.97) | 0.81 (0.39, 1.70) | 298 (24) |
| Swam in the sea, freshwater, outdoor hot tub, or pool | 60 (16) | 1.20 (0.87, 1.64) | 1.13 (0.79, 1.61) | 16 (17) | 1.25 (0.72, 2.18) | 0.83 (0.42, 1.63) | 228 (85) | 1.07 (0.74, 1.55) | 1.19 (0.79, 1.81) | 194 (14) |
| Treated drinking water at home (chlorine or UV filter) | 202 (83) | 1.18 (0.81, 1.71) | 1.10 (0.73, 1.66) | 56 (81) | 1.06 (0.57, 1.98) | 0.85 (0.43, 1.70) | 129 (51) | 1.01 (0.77, 1.32) | 1.18 (0.87, 1.59) | 704 (80) |
| Frequently wash hands before making food | 239 (77) | 1.13 (0.84, 1.52) | 1.09 (0.80, 1.49) | 58 (75) | 1.01 (0.59, 1.73) | 1.03 (0.57, 1.85) | 5 (2) | 0.61 (0.24, 1.56) | 0.79 (0.30, 2.10) | 936 (75) |
| Water supply for 1-19 houses vs baseline of 20+ houses | 38 (12) | 1.06 (0.73, 1.56) | 1.04 (0.68, 1.57) | 14 (18) | 1.62 (0.89, 2.96) | 1.75 (0.88, 3.47) | 44 (16) | 1.18 (0.83, 1.69) | 1.28 (0.86, 1.91) | 140 (12) |
| Work at a cafe, restaurant, or other serving place | 11 (3) | 1.04 (0.53, 2.05) | 0.86 (0.42, 1.75) | 1 (1) | 0.37 (0.05, 2.69) | 0.33 (0.04, 2.59) | 4 (1) | 0.61 (0.21, 1.73) | 0.79 (0.27, 2.33) | 41 (3) |
| Eating food made at a restaurant | 170 (51) | 1.00 (0.79, 1.27) | 1.17 (0.90, 1.54) | 41 (50) | 0.98 (0.63, 1.53) | 1.17 (0.69, 1.97) | 146 (83) | 1.24 (0.80, 1.91) | 1.24 (0.77, 2.02) | 711 (51) |
| Frequently wash hands before eating food | 134 (43) | 0.95 (0.74, 1.23) | 1.03 (0.78, 1.36) | 29 (36) | 0.69 (0.43, 1.11) | 0.92 (0.53, 1.58) | 127 (57) | 0.92 (0.69, 1.22) | 1.23 (0.89, 1.69) | 483 (45) |
| Frequently wash hands after bathroom | 302 (84) | 0.95 (0.69, 1.31) | 1.18 (0.82, 1.70) | 74 (80) | 0.71 (0.42, 1.21) | 1.09 (0.58, 2.05) | 181 (78) | 1.18 (0.84, 1.65) | 1.16 (0.81, 1.65) | 1158 (85) |
| Observed flies on food | 10 (3) | 0.94 (0.47, 1.90) | 1.13 (0.54, 2.38) | 5 (6) | 2.06 (0.79, 5.36) | 1.98 (0.64, 6.16) | 24 (10) | 0.89 (0.56, 1.40) | 0.81 (0.49, 1.32) | 41 (3) |
| Drinking tapwater away from home | 217 (65) | 0.93 (0.72, 1.20) | 0.94 (0.69, 1.27) | 66 (76) | 1.58 (0.95, 2.61) | 1.15 (0.60, 2.18) | 105 (46) | 1.06 (0.80, 1.41) | 1.08 (0.79, 1.48) | 916 (67) |
| Contact with dog or dog feces | 104 (32) | 0.91 (0.70, 1.18) | 0.92 (0.69, 1.21) | 31 (40) | 1.25 (0.78, 2.00) | 1.37 (0.81, 2.33) | 29 (10) | 0.96 (0.63, 1.46) | 1.14 (0.72, 1.80) | 442 (35) |
| Noticed something strange with home water | 8 (2) | 0.90 (0.41, 1.96) | 1.11 (0.49, 2.56) | 4 (4) | 1.74 (0.60, 5.01) | 2.61 (0.81, 8.43) | 54 (20) | 0.73 (0.53, 1.01) | 0.89 (0.63, 1.25) | 33 (2) |
| Live or work in retirement home, hospital, or other institution | 36 (10) | 0.88 (0.60, 1.29) | 1.00 (0.65, 1.52) | 7 (7) | 0.65 (0.30, 1.43) | 0.60 (0.23, 1.59) | 151 (61) | 0.79 (0.60, 1.04) | 0.84 (0.60, 1.18) | 158 (11) |
| Eating chicken | 163 (55) | 0.87 (0.67, 1.12) | 1.10 (0.83, 1.45) | 36 (51) | 0.72 (0.45, 1.16) | 0.80 (0.47, 1.35) | 73 (30) | 0.82 (0.61, 1.10) | 0.82 (0.59, 1.13) | 791 (59) |
| Eating food made on a grill | 82 (23) | 0.85 (0.64, 1.11) | 0.98 (0.73, 1.33) | 28 (30) | 1.22 (0.77, 1.93) | 1.31 (0.77, 2.22) | 84 (33) | 0.65 (0.49, 0.86) | 0.77 (0.57, 1.03) | 361 (26) |
| Frequently washes kitchen tools between use on raw meat and other food | 251 (83) | 0.84 (0.60, 1.18) | 0.77 (0.53, 1.12) | 53 (79) | 0.65 (0.35, 1.19) | 0.58 (0.29, 1.17) | 245 (93) | 0.68 (0.40, 1.15) | 0.84 (0.47, 1.50) | 1031 (85) |
| In contact with other animals or animal excrement | 44 (12) | 0.82 (0.58, 1.16) | 0.74 (0.51, 1.08) | 12 (14) | 0.93 (0.50, 1.74) | 0.85 (0.44, 1.66) | 10 (4) | 1.28 (0.63, 2.59) | 1.05 (0.50, 2.21) | 204 (15) |
| Frequently washes hands after contact with animals or birds | 119 (48) | 0.81 (0.62, 1.08) | 0.82 (0.60, 1.11) | 26 (42) | 0.63 (0.38, 1.06) | 0.57 (0.31, 1.04) | 39 (14) | 0.89 (0.61, 1.29) | 0.95 (0.63, 1.43) | 501 (53) |
| Other person in household abroad in the last four weeks | 49 (13) | 0.81 (0.58, 1.13) | 0.85 (0.59, 1.22) | 10 (10) | 0.61 (0.31, 1.18) | 0.62 (0.30, 1.26) | 104 (46) | 0.79 (0.59, 1.04) | 0.86 (0.63, 1.16) | 215 (16) |
| Eating cold cuts | 203 (63) | 0.79 (0.61, 1.02) | 0.78 (0.59, 1.02) | 58 (68) | 0.99 (0.62, 1.58) | 0.88 (0.52, 1.48) | 93 (50) | 0.89 (0.65, 1.21) | 0.91 (0.65, 1.29) | 946 (69) |
| Eating raw berries | 127 (37) | 0.76 (0.60, 0.98) | 0.87 (0.67, 1.13) | 43 (48) | 1.18 (0.77, 1.82) | 1.24 (0.77, 2.01) | 192 (82) | 0.71 (0.50, 1.03) | 0.72 (0.47, 1.08) | 601 (44) |
| Eating beef | 138 (45) | 0.75 (0.59, 0.96) | 0.82 (0.63, 1.08) | 34 (42) | 0.66 (0.42, 1.04) | 0.68 (0.41, 1.14) | 145 (61) | 0.73 (0.55, 0.97) | 0.75 (0.55, 1.02) | 714 (52) |
| Frequently wash hands after contact with raw meat | 214 (75) | 0.75 (0.55, 1.01) | 0.73 (0.53, 1.01) | 46 (75) | 0.75 (0.41, 1.36) | 0.77 (0.39, 1.50) | 198 (84) | 0.91 (0.62, 1.34) | 0.82 (0.54, 1.25) | 927 (80) |
| Drinking water from a dispenser | 46 (13) | 0.74 (0.53, 1.04) | 0.72 (0.49, 1.06) | 12 (13) | 0.71 (0.38, 1.32) | 0.58 (0.27, 1.22) | 262 (94) | 0.52 (0.29, 0.93) | 0.62 (0.32, 1.21) | 232 (17) |
| Eating eggs | 266 (82) | 0.73 (0.53, 1.01) | 0.79 (0.55, 1.14) | 74 (83) | 0.79 (0.44, 1.40) | 0.97 (0.50, 1.89) | 32 (12) | 0.78 (0.52, 1.16) | 0.73 (0.47, 1.12) | 1206 (86) |
| Visited a farm with livestock | 26 (7) | 0.71 (0.46, 1.10) | 0.62 (0.39, 0.99) | 13 (14) | 1.45 (0.79, 2.68) | 1.35 (0.69, 2.64) | 10 (4) | 0.45 (0.23, 0.87) | 0.50 (0.26, 0.99) | 135 (10) |
| Eating raw fruit | 335 (93) | 0.70 (0.44, 1.13) | 0.87 (0.51, 1.47) | 90 (94) | 0.79 (0.33, 1.86) | 0.86 (0.32, 2.32) | 168 (75) | 0.74 (0.53, 1.04) | 0.72 (0.51, 1.03) | 1332 (95) |
| Drinking water directly from river, stream, or lake | 11 (3) | 0.69 (0.36, 1.32) | 0.72 (0.36, 1.42) | 3 (3) | 0.73 (0.23, 2.38) | 0.94 (0.27, 3.31) | 34 (13) | 0.75 (0.51, 1.11) | 0.80 (0.52, 1.22) | 60 (4) |
| Eating ground meat | 270 (82) | 0.67 (0.49, 0.92) | 0.62 (0.43, 0.89) | 78 (91) | 1.47 (0.70, 3.10) | 0.99 (0.44, 2.19) | 39 (16) | 0.73 (0.51, 1.06) | 0.81 (0.55, 1.19) | 1206 (87) |
| Drinking tapwater at home | 359 (95) | 0.67 (0.38, 1.18) | 0.77 (0.40, 1.46) | 97 (99) | 3.06 (0.42, 22.48) | 2.68 (0.35, 20.73) | 8 (3) | 0.67 (0.32, 1.42) | 0.67 (0.31, 1.47) | 1362 (97) |
| Contact with wild birds or bird feces | 18 (5) | 0.63 (0.38, 1.05) | 0.74 (0.43, 1.25) | 8 (10) | 1.26 (0.59, 2.67) | 1.83 (0.81, 4.14) | 51 (21) | 0.52 (0.37, 0.72)** | 0.63 (0.44, 0.90) | 115 (8) |
| Eating lamb/mutton | 46 (13) | 0.62 (0.44, 0.87) | 0.72 (0.50, 1.03) | 7 (8) | 0.34 (0.15, 0.74) | 0.42 (0.18, 1.02) | 192 (78) | 0.55 (0.39, 0.77)* | 0.57 (0.39, 0.84) | 278 (20) |
| Eating turkey | 15 (4) | 0.62 (0.35, 1.08) | 0.58 (0.31, 1.10) | 1 (1) | - | CASE- | 13 (5) | 0.47 (0.26, 0.84) | 0.39 (0.21, 0.75) | 92 (7) |
| Live on a farm with livestock | 10 (3) | 0.59 (0.30, 1.17) | 0.59 (0.29, 1.20) | 6 (6) | 1.40 (0.59, 3.32) | 1.39 (0.52, 3.71) | 41 (16) | 0.61 (0.43, 0.87) | 0.62 (0.41, 0.92) | 62 (4) |
| Eating cured meats | 164 (50) | 0.57 (0.45, 0.73)*** | 0.60 (0.46, 0.78)** | 53 (61) | 0.88 (0.57, 1.37) | 0.95 (0.57, 1.58) | 111 (46) | 0.49 (0.37, 0.65)*** | 0.51 (0.38, 0.69)*** | 879 (64) |
| Eating meats purchased abroad | 53 (15) | 0.57 (0.42, 0.79)* | 0.61 (0.42, 0.88) | 12 (13) | 0.48 (0.26, 0.89) | 0.54 (0.24, 1.21) | 4 (1) | 0.32 (0.12, 0.88) | 0.31 (0.11, 0.89) | 324 (24) |
| Eating raw vegetables | 232 (71) | 0.50 (0.38, 0.65)*** | 0.59 (0.43, 0.80)* | 55 (67) | 0.41 (0.26, 0.67)* | 0.53 (0.29, 0.94) | 177 (72) | 0.53 (0.39, 0.72)** | 0.62 (0.44, 0.88) | 1159 (83) |
| Eating pork | 138 (46) | 0.49 (0.38, 0.63)*** | 0.48 (0.37, 0.64)*** | 34 (43) | 0.43 (0.27, 0.67)* | 0.44 (0.26, 0.73)+ | 14 (5) | 0.79 (0.44, 1.40) | 0.79 (0.42, 1.49) | 863 (63) |
| Eating salad | 198 (60) | 0.48 (0.37, 0.61)*** | 0.51 (0.38, 0.68)*** | 48 (59) | 0.45 (0.29, 0.71)* | 0.44 (0.25, 0.77) | 150 (60) | 0.48 (0.36, 0.64)*** | 0.52 (0.38, 0.72)** | 1053 (76) |
| Eating soft cheese | 65 (19) | 0.47 (0.35, 0.63)*** | 0.63 (0.46, 0.87) | 14 (15) | 0.36 (0.20, 0.63)* | 0.60 (0.31, 1.14) | 104 (47) | 0.52 (0.39, 0.69)*** | 0.50 (0.36, 0.68)*** | 464 (34) |
| Eating dried herbs | 203 (65) | 0.43 (0.33, 0.57)*** | 0.45 (0.33, 0.61)*** | 51 (65) | 0.43 (0.26, 0.69)* | 0.39 (0.22, 0.67)* | 32 (14) | 0.37 (0.25, 0.55)*** | 0.47 (0.31, 0.71)* | 1113 (81) |
| Eating fresh herbs | 45 (14) | 0.38 (0.27, 0.54)*** | 0.48 (0.33, 0.69)** | 13 (15) | 0.41 (0.23, 0.75) | 0.46 (0.23, 0.91) | 152 (65) | 0.43 (0.32, 0.59)*** | 0.47 (0.34, 0.65)*** | 409 (30) |
| Eating asparagus | 6 (2) | 0.37 (0.16, 0.86) | 0.34 (0.13, 0.88) | 0 (0) | - | CASE- | 6 (2) | 0.50 (0.21, 1.16) | 0.47 (0.18, 1.19) | 61 (4) |
| Drinking unpasturised milk | 2 (1) | 0.25 (0.06, 1.04) | 0.24 (0.06, 1.04) | 0 (0) | - | CASE- | 2 (1) | 0.33 (0.08, 1.41) | 0.34 (0.08, 1.46) | 30 (2) |
| Hedgehog in garden or neighbourhood | 1 (0) | - | CASE- | 0 (0) | - | CASE- | 1 (0) | - | CASE- | 3 (0) |
| Turtle, snakes, or other reptiles in household | 1 (0) | - | CASE- | 0 (0) | - | CASE- | 1 (0) | - | CASE- | 3 (0) |

*^#^All variables where CI does not contain 0 for at least one Salmonella serovar; Answers for all variables were not available from all participants; aOR: Adjusted for sex, age, number of people in household, county (dummy and education; Corrected for multiple testing; Significance indicators: 0 *** 0.001 ** 0.01*
